# Low to moderate alcohol consumption across two decades and subclinical atherosclerosis at age 60: findings from the Northern Sweden Västerbotten Intervention Programme—visualisation of atherosclerosis (VIPVIZA) study

**DOI:** 10.3389/fcvm.2025.1710165

**Published:** 2026-01-07

**Authors:** Albin Dahlin Almevall, Patrik Wennberg, Emma Nyman, Johan Hultdin, Mats Ramstedt, Anders Själander, Maria Wennberg

**Affiliations:** 1 Department of Public Health and Clinical Medicine, Umeå University, Umeå, Sweden; 2 Department of Diagnostics and Intervention, Umeå University, Umeå, Sweden; 3 Department of Medical Biosciences, Umeå University, Umeå, Sweden; 4 Department of Clinical Neurosciences, Karolinska Institutet, Stockholm, Sweden

**Keywords:** alcohol consumption, long-term, longitudinal, subclinical atherosclerosis, carotid plaque, carotid intima-media thickness

## Abstract

**Background:**

Alcohol consumption at low to moderate levels has long been debated in relation to cardiovascular risk, with inconsistent findings. Multi-decade cohort data with repeated exposure assessments are rare, especially in a northern Scandinavian population. This study aims to investigate associations between alcohol consumption at age 40, 50, and 60 and markers of subclinical atherosclerosis [carotid plaque and intima-media thickness (IMT)] at age 60 in a healthy below-risk-threshold alcohol-consuming cohort in Northern Sweden.

**Methods:**

Participants in the Visualisation of Asymptomatic Atherosclerotic Disease for Optimum Cardiovascular Prevention (VIPVIZA) trial, aged 60 and with alcohol data from the Västerbotten Intervention Programme (VIP) at 40, 50, and 60, with below-risk-threshold alcohol consumption (>0 to ≤100 g/week) (*n* = 1,014) were included. Alcohol intake data were collected via a food frequency questionnaire in VIP. Carotid plaque and IMT were assessed at age 60 at VIPVIZA baseline.

**Results:**

Mean weekly alcohol consumption for the study period was 26 g (±21.4 g), higher in men (37.5 ± 23.8 g) than in women (19.2 ± 16.3 g) and increasing over time in both sexes. At age 60, 49.6% had carotid plaque, and mean IMT was 0.77 mm (±0.15). No indication of associations was found between midlife alcohol consumption and carotid plaque in the total cohort [odds ratio (OR): 1.00, 95% confidence interval (CI): 0.99–1.01], men (OR: 1.00, 95% CI: 0.99–1.01), or women (OR: 0.99, 95% CI: 0.99–1.00) per gram increase of weekly alcohol intake. No associations were observed across consumption groups (>25 to ≤50, >50 to ≤75, >75 to ≤100 vs. >0 to ≤25 g/week).

**Conclusion:**

No association was found between self-reported midlife alcohol consumption and subclinical atherosclerosis at age 60 in the VIPVIZA baseline cohort. Results were consistent across sexes and intake levels, contributing to the evidence base used to guide primary prevention and public health recommendations.

## Graphical Abstract

**Figure:**
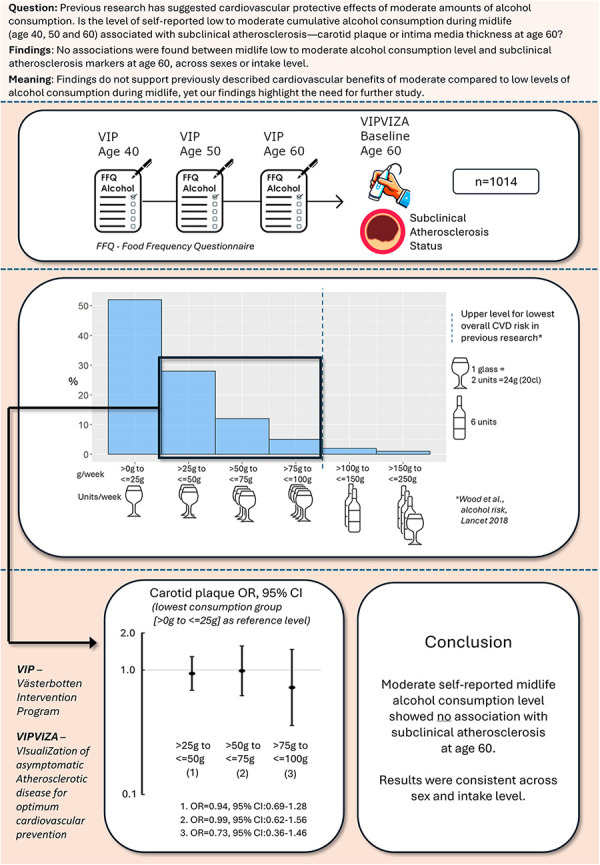

## Introduction

Atherosclerotic cardiovascular disease (CVD) is a leading cause of global mortality (1). Characterised by arterial dysfunction, inflammation, and dyslipidaemia, the onset of atherosclerosis involves multiple factors (2, 3). Subclinical signs of atherosclerosis include common carotid artery intima-media thickness (CIMT) and carotid atherosclerotic plaques, which have demonstrated predictive value for myocardial infarction and stroke (4–6). Atherosclerosis advances silently over decades and can, without prior symptoms (7), lead to CVD events such as myocardial infarction and stroke, with increasing prevalence with age (8, 9). Most myocardial infarctions and strokes are, however, attributed to highly modifiable factors (10, 11). The potential for significant CVD prevention by adopting a healthy lifestyle is evident, and adherence to such guidelines has been reported to be associated with a substantial reduction in coronary heart disease (12, 13) and ischaemic stroke incidence (14). Thus, lifestyle adjustments have been proposed as the primary approach to reducing the CVD burden (15). While not smoking, physical activity, healthy dietary patterns, and maintaining a healthy weight are generally accepted strategies for lowering cardiovascular risk, the role of low to moderate long-term alcohol consumption in relation to CVD risk remains contested (16). Although alcohol consumption is generally a well-established negative health factor and has been described as the seventh leading risk factor for death and disease-adjusted life years (DALYs) (17), there is also research suggesting that alcohol consumption could have a potential J-shaped relationship on CVD outcomes, with beneficial effects in lower quantities (<100 g/week) (18). For the over-40 age group, the theoretical minimum-risk-exposure alcohol level has been estimated at 0.11–1.87 standard drinks per day (19). Sex-specific differences have also been observed, with inverse associations between alcohol consumption and CIMT, but a positive association with carotid plaque in men—whereas no such associations were seen in women (20). Possible protective mechanisms of alcohol in moderate doses have been ascribed to short-term lowering effects on blood pressure (21); raised HDL cholesterol (22); and lower levels of C-reactive protein, interleukin-6, and interleukin-1 receptor antagonist (23). In addition, there is some support for improved insulin sensitivity (24). However, recent Mendelian randomisation studies using UK Biobank data suggest that even low levels of alcohol consumption, within current risk thresholds, may increase the risk of cardiovascular disease (25). In this study, we combine repeated self-reported alcohol consumption data from the Västerbotten Intervention Programme (VIP) population cohort of middle-aged men and women at ages 40, 50, and 60 with indicators of subclinical carotid atherosclerosis at age 60, utilising two of the most powerful CVD-predicting markers: carotid plaque and intima-media thickness (IMT).

## Aim

We aim to investigate the longitudinal associations between cumulative alcohol consumption at ages 40, 50, and 60 and markers of subclinical atherosclerosis—namely, carotid plaque presence and IMT—at age 60, in a Northern Sweden healthy midlife cohort with alcohol intake above zero yet below established risk thresholds.

## Materials and methods

### Cohort description

This study was conducted within VIP, a population-based health initiative targeting residents of Västerbotten County in Northern Sweden (26) and the Visualisation of Asymptomatic Atherosclerotic Disease for Optimum Cardiovascular Prevention (VIPVIZA) trial. The VIP programme invites participants to cardiovascular risk screening at primary care centres upon reaching age 40, 50, and 60, coupled with motivational interviewing aimed at preventing CVD and diabetes. The VIPVIZA trial is a multifaceted intervention that includes visual feedback on carotid atherosclerosis and a follow-up motivational call, as further detailed in previous publications (27).

**Figure 1:**
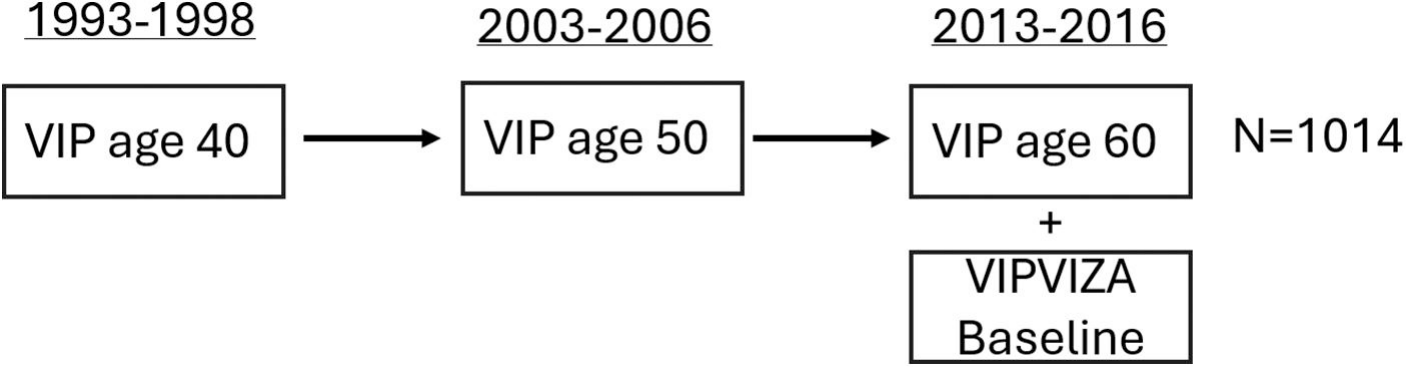
Study design overview illustrating the VIPVIZA baseline cohort and highlighting participants with historical data on alcohol consumption derived from VIP FFQs completed at ages 40, 50, and 60 included in this study. Carotid ultrasound measurements were conducted at age 60 (*n* = 1,014).

In this study, participants were selected from those who had enrolled in the VIPVIZA trial during their VIP visit at age 60 and had completed food frequency questionnaires (FFQs), including alcohol data, during earlier VIP visits at ages 40 (1993–1998), 50 (2003–2006), and 60 (2013–2016) (Figure 1). Carotid ultrasound examinations for the VIPVIZA baseline, which provided the outcome measures for the current study, took place between 29 April 2013 and 7 June 2016. Information on demographics, socioeconomic status, psychosocial factors, lifestyle, health, and dietary intake was gathered during VIP visits at ages 40, 50, and 60, with the age 60 visit in conjunction with the VIPVIZA baseline assessment. For this study, alcohol consumption data were derived from FFQs administered during VIP visits, curated by the Northern Sweden Diet Database (NSDD) (28). Reporting follows the STROBE guidelines for cohort studies (29).

### Subclinical carotid atherosclerosis

Carotid 2D B-mode ultrasound examinations were conducted using standardised protocol (30) by sonographers (biomedical analysts) specifically trained in carotid imaging at the Department of Clinical Heart Centre, Umeå University. Portable ultrasound equipment with a 7 MHz linear transducer (CardioHealth Station, Panasonic Healthcare Corporation of North America, Newark, NJ, USA) was used. Bilateral, real-time automatic measurements of CIMT were obtained along a 1 cm segment of the far wall in the distal common carotid arteries at the end diastole, using insonation angles of 120° and 150° on the right side and 210° and 240° on the left. The ultrasound system automatically provided the insonation angles (6). The presence of atherosclerotic plaque was assessed bilaterally across the entire carotid artery in accordance with the Mannheim consensus (31). Inter-operator reproducibility for plaque detection yielded a kappa value of 0.70 [95% confidence interval (CI): 0.60–0.80] (32), while inter-operator variability for CIMT had an intraclass correlation coefficient of 0.95 (33). In this study, plaque presence was defined as any detected plaque, regardless of size or location.

### FFQ and alcohol consumption

Alcohol intake was calculated from validated, self-reported semi-quantitative FFQs completed by participants at VIP visits at ages 40, 50, and 60 (34). In the FFQ, consumption of food items during the last year was reported through nine alternatives: *never*, *a few times a year*, *1–3 times per month*, *once a week*, *2–3 times a week*, *4–6 times a week*, *once a day*, *2–3 times a day*, and *four or more times a day*. Grams/day of different food items were calculated by multiplying the reported frequency by the portion size. Five FFQ items were used to calculate alcohol intake: *light beer* [2.3% alcohol by volume (a.b.v.)], *medium beer* (3.5% a.b.v.), *strong beer* (5.6% a.b.v.), *wine* (combination of red wine 12% a.b.v. and white wine 12% a.b.v.), and *spirits* (40% a.b.v.). To facilitate interpretation and comparisons, alcohol intake was recalculated to grams/week for this study, which aligns with commonly reported amounts in existing literature. In addition, for illustrative reasons, alcohol consumption is expressed in standard units according to the Swedish definition “*standard glas*”, which is often used in health recommendations and public guidelines, to further increase the relevance and accessibility of the findings. One unit of alcohol, according to this specific definition, corresponds to 12 g, while grams/unit can vary by country and context.

### Covariates

The covariates included sex (male, female); smoking status (current, former, occasional, former occasional, or never); snus (smokeless tobacco placed under the upper lip) use (current user, former user, or never user); physical activity level [inactive (little to no regular exercise or active commuting), moderately active (some exercise or active commuting at least a few times per week), or active (regular exercise ≥2–3 times/week or consistent active commuting over longer distances)]; educational attainment [basic education (up to 9 years of schooling), middle (10–12 years), or high (≥13 years)]; use of blood-pressure- or lipid-lowering medications; self-reported diabetes; impaired glucose tolerance (IGT), defined as oral glucose tolerance test (OGTT) ≥8.9; body mass index (BMI), calculated as weight in kilograms divided by height in metres squared; and total cholesterol. CVD family history, specified as a history of myocardial infarction or stroke before age 60 in first-degree relatives, was also incorporated. Due to a limited number of cases, blood-pressure- and lipid-lowering medication use at age 40 was combined with CVD family history to form a “CVD risk group”, which was included as a covariate in Model 3 (Table 1) analysis. Dietary pattern was also used as a covariate based on the Healthy Diet Score (HDS), which does not include alcohol and has been described in detail in the current cohort in another study (35). The HDS range from 0, representing the lowest adherence, to 24, representing the highest. All covariates were recorded at age 40, except for CVD family history, which was self-reported at age 60. A framework for the adjustment strategy and proposed causal pathways is illustrated in a directed acyclic graph (DAG) in Supplementary Material.

**Table 1:** Regression models in the analysis.

Crude associations	Model 1: primary	Model 2: primary + lifestyle	Model 3: primary + lifestyle + potential mediators
NA	Sex	Sex	Sex
	Educational level	Educational level	Educational level
	CVD family history	CVD family history	CVD family history
		Smoking	Smoking
		Snus use	Snus use
		Physical activity	Physical activity
		Diet (Healthy Diet Score)	Diet (Healthy Diet Score)
			SBP
			BMI
			Diabetes/IGT
			Cholesterol
			CVD preventive medication

CVD, cardiovascular disease. SBP, systolic blood pressure. BMI, body mass index. IGT, impaired glucose tolerance.

The table displays covariates included in each regression model.

### Exclusion

Of the 3,532 participants in the VIPVIZA baseline, 2,278 were of age 60 and were considered for inclusion in the present study. Of these, 1,049 had missing information on alcohol consumption at age 40, 50, or 60. Participants with a Cut down, Annoyed, Guilty, and Eye-opener (CAGE) alcohol use disorder (36) score of ≥2 (*n* = 174) were excluded. Also, people who reported abstaining at all ages (40, 50, and 60) (*n* = 22) and those with a total study period mean above-risk-threshold alcohol consumption (100 g/week) (*n* = 29) were not included in the analysis (Supplementary Material). Exclusions were motivated by difficulties controlling for potential confounding factors specifically linked to alcohol abstinence or high consumption that may be associated with alcohol abuse, chronic disease, or health risk behaviour.

### Statistical methods

#### Mean reported alcohol intake for ages 40, 50, and 60 and subclinical carotid plaque association

Binary logistic regression was used to assess the association between mean alcohol consumption (grams/week) at ages 40, 50, and 60 and the presence of carotid plaque. Analyses were conducted for the total study cohort, as well as for groups stratified by sex. The significance of associations was evaluated using Wald tests and odds ratios (ORs) with 95% CIs. Model fit was assessed using the Hosmer–Lemeshow test for goodness of fit. Three models with the stepwise inclusion of covariates were used to study the effects of adjustments, and a non-adjusted model was used to assess crude associations. Model 1 included key confounders, model 2 incorporated additional lifestyle-related confounders, and model 3 further adjusted for potential mediators (Table 1).

#### Alcohol in categories and binary plaque/CIMT outcomes

Binary logistic regression was used to assess and visualise associations between alcohol consumption levels, as described by Wood et al. (18) (see first paragraph in the Results section), and the binary outcome (carotid plaque) in the total cohort. Each group was compared with the reference category, which was the lowest-alcohol-consumption group (>0–≤25 g/week). Results were presented in a forest plot with ORs and 95% CIs, adjusted for model 2 covariates (Table 1).

#### Non-linear associations alcohol/CIMT

A series of ordinary least squares (OLS) regression models was fitted to examine the association between mean weekly alcohol consumption at ages 40, 50, and 60 and CIMT. First, an unadjusted model was estimated using OLS regression. To account for potential non-linearity in the association, restricted cubic splines with four knots, placed at the 5th, 35th, 65th, and 95th percentiles, were applied to the alcohol-consumption variable. The final adjusted model included model 2 (Table 1) covariates. Model fit was assessed using analysis of variance (ANOVA). Predicted values of CIMT across the alcohol consumption range (0–100 g/week) were obtained using the fitted model, with CIs calculated using mean-based prediction intervals. A visualisation of the predicted association was created, displaying the estimated regression line with a shaded confidence interval. Total-cohort and sex-stratified analyses were carried out. R packages rms, Hmisc, mice, ggplot2, and scales were utilised.

#### Missing values

For exposure and outcome variables, only complete cases were included. Missing covariate data were replaced by multiple imputation using linear and logistic regression in SPSS. The results were pooled across five imputed datasets. In R, multiple imputation by chained equations (MICE) was used to handle missing data. Predictive mean matching (PMM) was applied with five imputed datasets.

#### Significance level

A *p*-value <0.05 was considered statistically significant in the analyses.

#### Residual diagnostics

To assess model fit and identify potential outliers, residuals from all binary logistic regression models were examined. Cook's distance, leverage values, Pearson residuals, and deviance residuals for all models were evaluated. No observations exhibited problematic values, with all residuals remaining well within acceptable limits. The analysis indicated that there were no influential outliers or issues with model fit related to residual distributions.

#### Sensitivity analysis

A five-step sensitivity analysis was performed by (1) including abstainers and cases with a mean alcohol consumption of ≥100 g/week and CAGE ≥2; (2) comparing the total cohort as two groups of extreme values, defined as cases that reported either above- or below-median alcohol consumption consistently at ages 40, 50, and 60, in a χ^2^ test; (3) analysing differences in CIMT between groups of extreme values (consistent high/low alcohol consumption at ages 40, 50, and 60) specifically for women, using ANOVA; (4) analysing alcohol consumption and association with plaque presence constructed as an ordinal variable (no plaque found, plaque on one side, plaque on both sides) in ordinal logistic regression; and, finally, (5) analysing only alcohol consumption at age 40 and its associations with carotid plaque presence at age 60. The sensitivity analysis results were considered in the interpretations of results but are not reported in detail in this article.

#### Analysis software

Statistical analyses were carried out using SPSS Statistics, version 29.0 (IBM SPSS Inc., IBM Corp, Armonk, NY, USA), and R, version 4.4.3.

#### Visualisations and illustrations

The R ggplot2 package was used to visualise the sex-stratified alcohol-consumption distribution with violin plots and to create an overlapping histogram. SPSS was used to create box plots. DAGitty version 3.1 was used to construct and illustrate the DAG, and GIMP version 2.10.38 was used to edit figures.

## Results

### VIP cohort: comparison and context

The VIP cohort aged 40, 50, and 60 is a low-alcohol-intake-reporting cohort compared to large cohorts. Wood et al. (18) presented alcohol consumption risk thresholds in three large cohorts—Emerging Risk Factor Collaboration (ERFC), European Prospective Investigation into Cancer and Nutrition-Cardiovascular Disease (EPIC-CVD), and the UK Biobank—in which alcohol consumption was categorised into groups of (1) >0 to ≤25, (2) >25 to ≤50, (3) >50 to ≤75, (4) >75 to ≤100, (5) >100 to ≤150, (6) >150 to ≤250, (7) >250 to ≤350 and (8) >350 g/week. According to this categorisation, 51.6% of the VIP cohort (total abstainers not included), reported the lowest intake category over the study period of 40, 50, and 60 years, with less than the equivalent of around a large (20 cL) glass of wine/week. Another 28.7% reported being in the second lowest category, with a maximum of around two glasses; 12.5% were in the third category, with the equivalent of three glasses; and 4.7% reported four glasses/week (with wine used as an example of an alcoholic beverage). Only 2.4% reported higher consumption. Alcohol consumption distribution means in VIP for ages 40, 50, and 60 (consistent abstainers not included) are illustrated in Supplementary Material as an overlapping histogram with the EPIC-CVD cohort for comparison. Note that the VIP 1992–1996 cohort alcohol consumption data *are included* in the EPIC-CVD cohort presented by Wood et al. (18).

### Participant characteristics

Participant characteristics are presented in Table 2, stratified by carotid plaque presence at age 60 and covariate distribution at age 40, which were used in the analysis models. Table 3 describes age 40 characteristics and atherosclerosis markers at age 60 by mean weekly alcohol consumption, divided into four level groups [as described by Wood et al. (18), up to ≤100 g/week] over the study period.

**Table 2:** Included participants’ characteristics at VIP age 40 and subclinical carotid plaque at age 60 VIPVIZA baseline (*n* = 1,014).

Characteristics	Carotid plaque present (*n* = 511)	No carotid plaque (*n* = 503)
Male	218 (57.7)	160 (42.3)
Female	293 (46.1)	342 (53.9)
Alcohol intake, g/week	26.73 ± 21.41	25.25 ± 21.32
Serum cholesterol, mmol/L	5.61 ± 1.10	5.29 ± 1.02
Missing	1	2
Body mass index, kg/m^2^	24.79 ± 3.55	24.76 ± 3.64
Glucose 2 h value, mmol/L	6.29 ± 1.24	6.36 ± 1.21
Missing	24	19
Systolic BP, mmHg	120.99 ± 13.14	117.18 ± 11.55
Missing	1	2
Diastolic BP, mmHg	76.09 ± 9.48	74.12 ± 9.08
Missing	1	2
Healthy Diet Score	11.02 ± 11.02	11.62 ± 3.75
Smoker	119 (23.5)	76 (15.3)
Former smoker	106 (20.9)	98 (19.7)
Never smoker	213 (42.1)	253 (50.9)
Occasional smoker	24 (4.7)	19 (3.8)
Former occasional smoker	44 (8.7)	51 (10.3)
Missing	5	6
Snus user	67 (13.4)	52 (10.5)
Former snus user	53 (10.6)	38 (7.7)
Not using snus	381 (76)	403 (81.7)
Missing	10	10
CVD heredity (at age 60)	118 (23.2)	110 (21.9)
Missing	2	—
BP-lowering medication	10 (2.0)	9 (1.8)
Lipid-lowering medication	<3	<3
Education: Basic (9 years)	74 (14.5)	52 (10.4)
Education: Middle (10–12 years)	309 (60.6)	296 (59.1)
Education: High (≥13 years)	127 (24.9)	153 (30.5)
Missing	1	2
Diabetes	<3	<3
Missing	2	—
Physical activity: inactive	88 (17.3)	75 (14.9)
Physical activity: moderate	355 (69.9)	355 (70.6)
Physical activity: active	67 (13.1)	73 (14.5)
Missing	1	—

Values are presented as mean ± standard deviation (SD) for continuous variables and as *n* (%) for categorical variables. Values <3 not detailed for confidentiality.

**Table 3:** Included participants characteristics’ at VIP age 40 and mean alcohol consumption/week for ages 40, 50, and 60 in first four groups (>0 to ≤100 g/week) according to Wood et al. (18), including subclinical atherosclerosis outcomes at age 60 (*n* = 1,014).

Characteristics	>0 to ≤25 g (*n* = 594)	>25 to ≤50 g (*n* = 275)	>50 to ≤75 g (*n* = 107)	>75 to ≤100 g (*n* = 38)
Male	135 (22.7)	131 (47.6)	79 (73.8)	33 (86.8)
Female	459 (77.3)	144 (52.4)	28 (26.2)	5 (13.2)
Carotid plaque present	296 (49.8)	136 (49.5)	60 (56.1)	19 (50)
CIMT	.76 ± .15	.76 ± .14	.79 ± .18	.82 ± .18
Serum cholesterol, mmol/L	5.45 ± 1.08	5.43 ± 0.99	5.48 ± 1.18	5.53 ± 1.31
Missing	1	—	1	1
Body mass index, kg/m^2^	24.95 ± 3.78	24.53 ± 3.43	24.47 ± 2.75	24.65 ± 2.22
Glucose 2 h value, mmol/L	6.50 (1.21)	6.28 ± 1.14	5.73 ± 1.28	5.68 ± 1.03
Missing	27	11	2	3
Systolic BP, mmHg	119.17 ± 12.51	118.81	119.67 ± 11.96	118.66 ± 10.04
Missing	2	—	1	—
Diastolic BP, mmHg	75.28 ± 9.21	74.78 ± 9.51	75.27 ± 9.09	74.55 ± 10.80
Missing	2	—	1	—
Healthy Diet Score	11.43 ± 3.92	11.35 ± 3.56	10.80 ± 3.36	10.87 ± 2.82
Smoker	117 (19.9)	47 (17.3)	26 (24.3)	5 (13.2)
Former smoker	120 (20.4)	55 (20.3)	22 (20.6)	7 (18.4)
Never smoker	284 (48.4)	121 (44.6)	40 (37.4)	21 (55.3)
Occasional smoker	18 (3.1)	17 (6.3)	7 (6.5)	<3
Former occasional smoker	48 (8.2)	31 (11.4)	12 (11.2)	4 (10.5)
Missing	7	4	—	—
Snus user	44 (7.6)	46 (17.2)	23 (21.7)	6 (15.8)
Former snus user	33 (5.7)	29 (10.8)	24 (22.6)	5 (13.2)
Not using snus	505 (86.8)	193 (72)	59 (55.7)	27 (71.1)
Missing	12	7	1	—
CVD heredity (at age 60)	142 (23.9)	52 (19.0)	28 (26.2)	6 (16.2)
Missing	—	1	—	1
BP-lowering medication	10 (1.7)	6 (2.2)	<3	0
Lipid-lowering medication	<3	<3	0	0
Education: Basic (9 years)	80 (13.5)	29 (10.5)	11 (10.3)	6 (15.8)
Education: Middle (10–12 years)	356 (61.8)	166 (60.4)	54 (50.5)	20 (52.6)
Education: High (≥13 years)	146 (24.7)	80 (29.1)	42 (39.3)	12 (31.6)
Missing	3	—	—	—
Diabetes	3 (.5)	0	0	0
Missing	1	1	—	—
Physical activity: Inactive	97 (16.4)	45 (16.4)	16 (15)	5 (13.2)
Physical activity: Moderate	420 (70.7)	192 (69.8)	73 (68.2)	25 (65.8)
Physical activity: Active	76 (12.8)	38 (13.8)	18 (16.8)	8 (21.1)
Missing	1	—	—	—

Values are presented as mean ± standard deviation (SD) for continuous variables and as *n* (%) for categorical variables. Values <3 not detailed for confidentiality.

### Alcohol consumption

#### Cumulative alcohol consumption distribution

**Figure 2:**
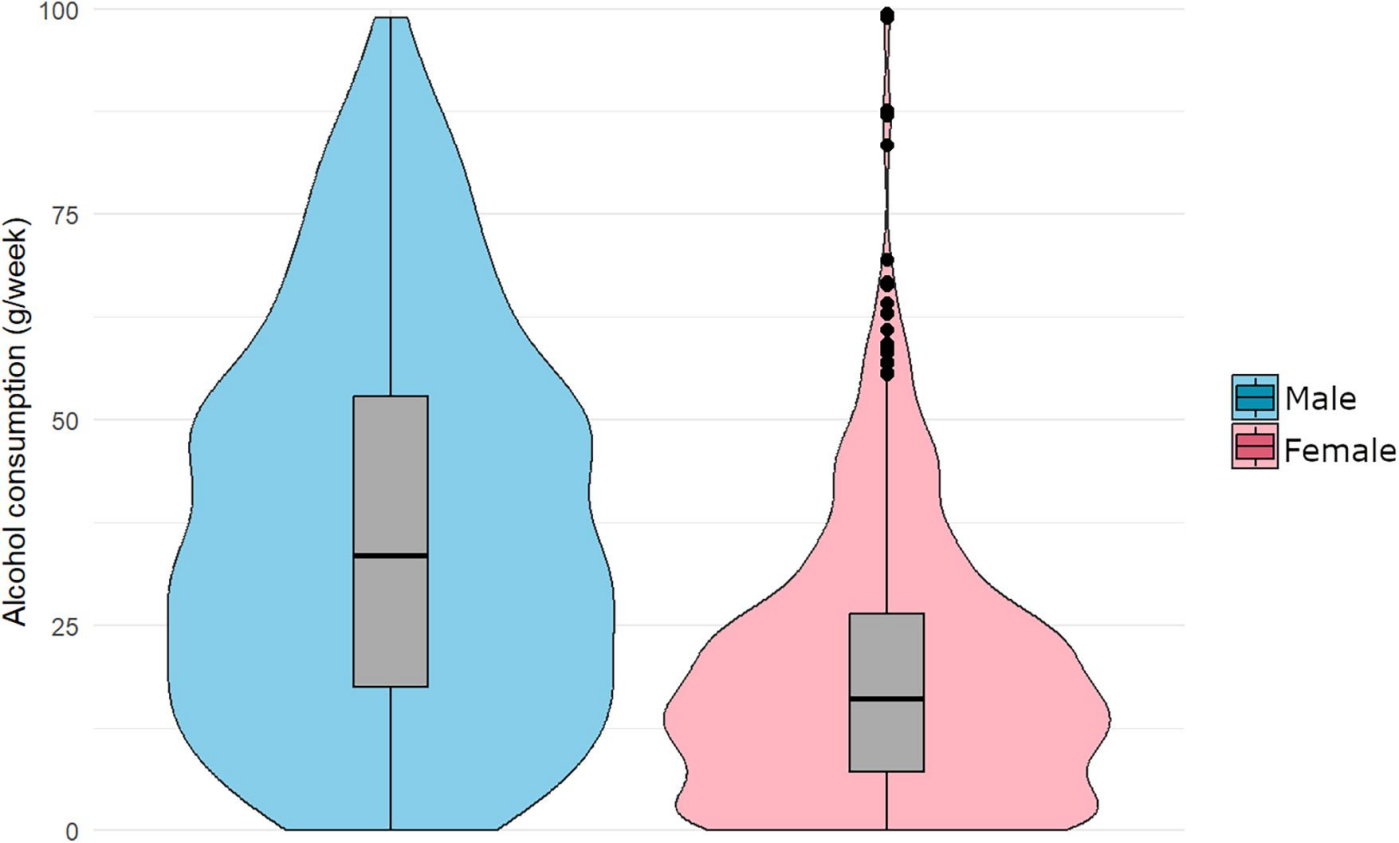
Distribution of mean alcohol consumption (grams/week) across ages 40, 50, and 60, stratified by sex. Violin plots illustrate the kernel density estimation, while boxplots represent the IQR. The median is marked by the horizontal line inside the box. “Extreme” cases (*n* = 23) are shown as points in the plot, defined as values greater than Q3 + 1.5 × IQR (*n* = 1,014).

The mean weekly alcohol consumption (grams/week), based on cumulative intake for ages 40, 50, and 60, was higher in men (37.5 g/week) than in women (19.2 g/week). Furthermore, the median consumption was 33.5 g/week for men and 16.0 g/week for women. The interquartile range (IQR) was broader in men (17.4–52.9 g/week) than in women (7.15–26.3 g/week), indicating greater variability in alcohol-consumption patterns. In women, 23 high-consumption outliers were identified according to Tukey's rule (1.5 × IQR below/above Q1, Q3), whereas no outliers were observed among men (Figure 2).

#### Alcohol consumption over the study period

The mean FFQ-reported alcohol in the included cohort slightly increased throughout the study period. At age 40, women reported a mean weekly alcohol consumption of 17.1 g/week, while men reported 34.9 g/week. At age 50, women reported 19.1 g/week and men, 38.3 g/week. Consumption peaked at age 60 for both sexes, with women reporting 21.3 g/week and men reporting 39.2 g/week (Supplementary Material).

### Cumulative alcohol at ages 40, 50, and 60 and carotid plaque at age 60

The interaction term for sex and alcohol introduced to the models was significant for the crude associations but not for models 1–3; however, a sex-stratified analysis was carried out, with results presented here. The Hosmer–Lemeshow test indicated good model fit for all models (*p* > 0.05 for all comparisons). The results with ORs and CIs are reported in Table 4.

**Table 4:** Total mean alcohol consumption for ages 40, 50, and 60 and plaque outcome at age 60.

Model	Alcohol (g/week) B	SE (B)	Exp(B) (OR)	95% CI for Exp(B)
Total cohort (*n* = 1,014)				
Crude	−0.005	0.004	0.995	0.986–1.004
Model 1	0.000	0.003	1.0	0.993–1.006
Model 2	−0.001	0.003	0.999	0.993–1.006
Model 3	0.001	0.004	1.0	0.993–1.007
Male (*n* = 378)				
Crude	−0.002	0.004	0.998	0.990–1.007
Model 1	0.004	0.002	0.998	0.995–1.002
Model 2	0.003	0.002	0.997	0.993–1.000
Model 3	0.003	0.002	0.998	0.994–1.002
Female (*n* = 636)				
Crude	0.000	0.005	1.000	0.990–1.009
Model 1	0.001	0.002	1.001	0.997–1.005
Model 2	−0.001	0.002	0.999	0.995–1.004
Model 3	−0.001	0.002	0.999	0.995–1.004

Associations between total mean alcohol consumption (g/week) at ages 40, 50, and 60 and carotid plaque outcome at age 60 are presented for the total cohort and stratified by sex. Binary logistic regression models were used to estimate ORs and 95% CIs. The models include the following: crude (unadjusted), model 1 [main confounders (sex, educational level, CVD family history)], model 2 [main confounders + lifestyle factors (smoking, snus use, physical activity, diet)], and model 3 [main confounders + lifestyle factors + potential mediators (systolic blood pressure, BMI, diabetes, cholesterol, preventive medication)] (Table 1). Regression coefficients (B) and standard errors (SE) are reported.

### Alcohol consumption levels and carotid plaque

**Figure 3:**
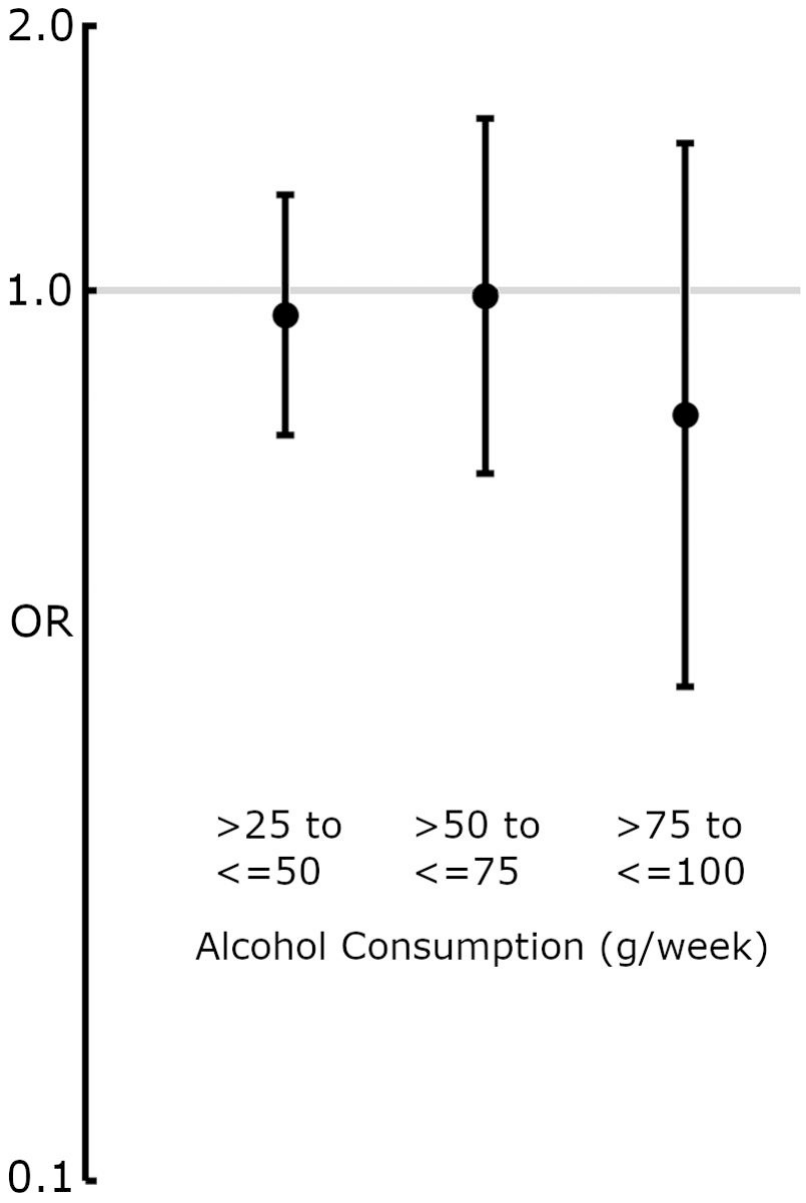
Forest plot displaying OR and 95% CIs for the association between alcohol consumption (grams/week) and the presence of carotid plaque at age 60. The lowest-alcohol-consumption reporting group (>0 to ≤25 g/week) was used as a reference. ORs were derived from binary logistic regression models adjusted for model 2 covariates (Table 1). None of the alcohol-consumption groups showed a significant association with carotid plaque presence.

In an analysis of total-cohort alcohol consumption in grams/week in groups corresponding with those described by Wood et al. (18), the lowest-alcohol-consuming group (>0 to ≤25 g, *n* = 594) was used as a reference for the higher consuming groups. None of the groups displayed significant probability of higher or lower presence of carotid plaque at age 60 (>25 to ≤50 g, OR: 0.94, 95% CI: 0.69–1.28, *n* = 275; >50 to ≤75 g, OR: 0.99, 95% CI: 62–1.56, *n* = 107; >75 to ≤100 g, OR: 0.73, 95% CI: 0.36–1.46, *n* = 38) compared with the lowest reporting group (Figure 3).

### Alcohol and CIMT: non-linear associations

A linear regression model was fitted to examine the association between mean weekly alcohol consumption at ages 40, 50, and 60 and CIMT, adjusting for model 2 (Table 1) covariates. In the unadjusted model, a 1-unit (grams/week) increase in alcohol consumption was associated with a 0.016 mm increase in CIMT (*p* = 0.006, 95% CI: 0.004–0.029). However, in the adjusted model using restricted cubic splines with four knots, no statistically significant association was observed (*p* = 0.48). The model explained a small proportion of variance (adjusted *R*^2^ = 0.04), suggesting that alcohol consumption accounts for a small portion of the variability in CIMT. In men, the unadjusted model showed no significant association between mean weekly alcohol consumption and CIMT (*p* = 0.64). Similarly, in the adjusted model, the overall effect of alcohol consumption remained non-significant (*p* = 0.37). The test for non-linearity did not indicate significant curvature in the relationship (*p* = 0.21), suggesting no evidence of a non-linear association. In women, the unadjusted model suggested a weak positive association between alcohol consumption and CIMT (*β* = 0.016, SE = 0.006, 95% CI: 0.004–0.029, *p* = 0.03). However, after adjusting for model 2 covariates, the association between alcohol consumption and CIMT was no longer significant (*p* = 0.06). The test for non-linearity remained non-significant (*p* = 0.22), indicating no evidence of a non-linear relationship (Supplementary Material).

## Discussion

This study examined cumulative alcohol consumption at ages 40, 50, and 60 in relation to carotid plaque and intima-media thickness at age 60 in the northern Sweden VIP/VIPVIZA cohort. Alcohol intake increased modestly over the 20-year period yet remained low compared with other large cohorts. We found no statistically significant associations between cumulative alcohol consumption and either carotid plaque or IMT in men or women.

### Low alcohol in the cohort

Alcohol consumption was low but slightly increased over midlife in both sexes. The amounts are below the amounts reported by other cohorts used herein for comparison [EPIC-CVD, ERFC, and UK Biobank (18)] and the current Swedish national average consumption of 11 units/week (37). In 2002, Sieri et al. (38) published a study describing alcohol consumption patterns, recruiting participants from the EPIC-CVD cohort in 10 countries. Between 1995 and 1998, a total of 35,955 participants between the ages of 35 and 74 were specifically interviewed about their alcohol intake amounts in the last 24 h, frequency, time of consumption, and type of beverage, with data being adjusted for weekdays and seasons. Of these, 1,344 were recruited from the VIP part of the EPIC-CVD cohort, which means that some of the participants in the present study could potentially have been included during their age-40 VIP visit, when they filled out their first FFQ. The 2002 Sieri et al. (38) study specifically described aspects of alcohol-consumption patterns in the aforementioned selection of VIP and other contemporary European settings; therefore, it can be related to and help inform the present study. One notable result of that study was that participants recruited from the VIP had the lowest reported alcohol consumption of all the EPIC-CVD sites, which seems to be in line with our findings.

### Population, alcohol type, and drinking pattern differences

The results of this study are specific to its context, culture, and era of data collection. Thus, the results might not be directly transferable to other settings that may have very different conditions, such as drinking patterns, diets, and genetics. For example, drinking patterns in the VIP were more concentrated around weekends, in comparison with other European settings, in which drinking is more spread out throughout the week (38). Concentrated drinking might have its own unique links to atherosclerosis development and other health outcomes compared with drinking small amounts daily, often primarily in connection to a meal. Furthermore, the type of alcoholic beverage is considered important to health outcomes. For example, in a longitudinal study following men since the 1960s in Zutphen, Netherlands, those consuming up to 20 g of wine/day had a CVD outcome hazard ratio (HR) of 0.61 (95% CI: 0.41–0.89) compared with those consuming corresponding amounts of beer (HR 0.82, 95% CI: 0.60–1.12) or spirits (HR 0.92, 95% CI: 0.63–1.34). Among the VIP participants described by Sieri et al. in 2002 (38), only 45%–50% of the alcoholic beverages consisted of wine, compared with Mediterranean study centres, where up to 94% of beverages consumed were wine. This information and our findings motivate nuancing beyond alcohol amounts in discussions about links between alcohol and CVD outcomes, as well as the need to be context-specific and cautious when generalising associations that in fact may differ significantly between populations.

### Sex differences

There was a slight, albeit not statistically significant, positive association between higher levels of alcohol consumed and CIMT in women; however, relatively few women reported alcohol consumption in the upper end of the studied alcohol consumption amounts. Furthermore, the sensitivity analysis of alcohol consumption in women and CIMT indicated no further evidence of such associations. We note at least three major differences between the sexes in both exposure and outcomes that motivate careful consideration in interpretations of results. First, women's alcohol consumption was about half that reported by men. The lower alcohol consumption among women in our cohort is in line with previously described sex differences in relation to total alcohol consumption and pharmacokinetics. Women generally have a lower total body water volume, in combination with a higher proportion of body fat, which results in higher blood alcohol concentrations for any given alcohol dose compared to men (39). These physiological differences could provide at least part of the explanation for the observed sex differences in consumption. Second, there were major differences in the type of alcoholic beverage consumed, which is quite possibly more beer based in men and wine based in women, as described by Sieri et al. (38) in VIP. This difference may affect atherosclerosis outcomes, as discussed in a previous paragraph. Third, there were differences in incident atherosclerosis at age 60 (women 46.1%, men 57.7%).

### Self-reporting of alcohol, compared with other ways of measuring

This study used FFQ-based reports of alcohol consumption during a normal week. Another approach to measure alcohol consumption is the use of biomarkers, where phosphatidyl ethanol (PEth) has been shown to have high sensitivity and specificity for detecting alcohol intake. PEth forms only in the presence of ethanol (40). Self-reports of alcohol consumption are known to be biased, and contextual factors are sensitive to self-reports. For example, in a cohort with chronic liver disease, only 33.7% of alcohol self-reports were consistent with PEth (41). At the same time, however, self-reports such as the AUDIT C have been described as aligning well with PEth in a general population (42). An issue with PEth is that it cannot discern differences between alcoholic beverage types. Thus, all forms of alcohol consumption measures have their own specific strengths and weaknesses and self-reports are still frequently used for feasibility reasons. All single markers are limited on their own. Future studies assessing alcohol exposure may benefit from a multi-marker approach, combining traditional biomarkers with markers such as metabolomic signatures, beverage-specific compounds, lipid or liver markers and self-reported intake to achieve a more stable, sensitive, and specific measure of alcohol consumption (43).

## Strengths and limitations

There are possible contributors to reported levels being low compared to other cohorts. As the VIP is a population-level intervention aiming to reduce health risk factors, and alcohol is one of the factors targeted, those who chose to participate in VIP may have lower alcohol consumption compared to a truly random sample from the general population. Further, self-reported alcohol consumption is prone to under-reporting issues; however, due to feasibility reasons, it remains a common reporting method and is what much of the current alcohol–CVD association research is based on. The highest alcohol consumption group was small, which limits statistical power and reduces the precision of estimates for this subgroup. A strength of this study compared to many other studies is that alcohol consumption was reported at repeated time points within the cohort, which should provide a sort of cumulative stability in the exposure analysed. The longitudinal design did not allow for adjusting for confounders at all time points in a methodologically feasible manner, and we chose to control for covariates at age 40. Furthermore, there may be differences between alcoholic beverages and their respective associations with atherosclerosis. Due to the low amounts of alcohol reported, we chose to investigate alcohol as the sum of alcoholic beverages. Although the atherosclerosis markers used in this study are strong predictors of CVD, and combining them with additional vascular or cardiac markers could offer complementary insights in future alcohol–CVD research.

## Conclusion

We found no association between cumulative self-reported alcohol consumption at ages 40, 50, and 60 and subclinical atherosclerosis markers (carotid plaque and IMT) at age 60 in a healthy, below-risk-threshold alcohol consumption population, in the Northern Sweden VIPVIZA baseline cohort. This lack of association remained consistent when the data were analysed separately by sex, across groups stratified by alcohol consumption levels. Participants reported low and slightly increasing alcohol intake throughout midlife, with men consistently reporting nearly twice the amount reported by women. The findings contribute to the evidence base used to inform primary care–based health dialogues and other prevention efforts by providing updated evidence on how low to moderate midlife alcohol consumption relates to late midlife subclinical atherosclerosis.

## Data Availability

The datasets presented in this article are not readily available because although access to individual-level data can be provided for research purposes, it is restricted by laws regarding the privacy of research. Requests to access the datasets should be directed to the Section of Biobank and Registry Support at Umeå University (contact: info.brs@umu.se).
