# Supplementary material for: Low to moderate alcohol consumption across two decades and subclinical atherosclerosis at age 60: findings from the Northern Sweden Västerbotten Intervention Programme—visualisation of atherosclerosis (VIPVIZA) study
Source: Front Cardiovasc Med. 2026 Jan 7;12:1710165. doi: 10.3389/fcvm.2025.1710165 (PMC12819816; doi:10.3389/fcvm.2025.1710165)
Supplement: Supplementary file 1 [file Datasheet1.pdf]

Supplementary Material

The supplementary material contains supporting figures related to the methodological framework and study data. This includes a directed acyclic graph (DAG), participant exclusion flowchart, distribution plots and exposure-outcome visualisations.

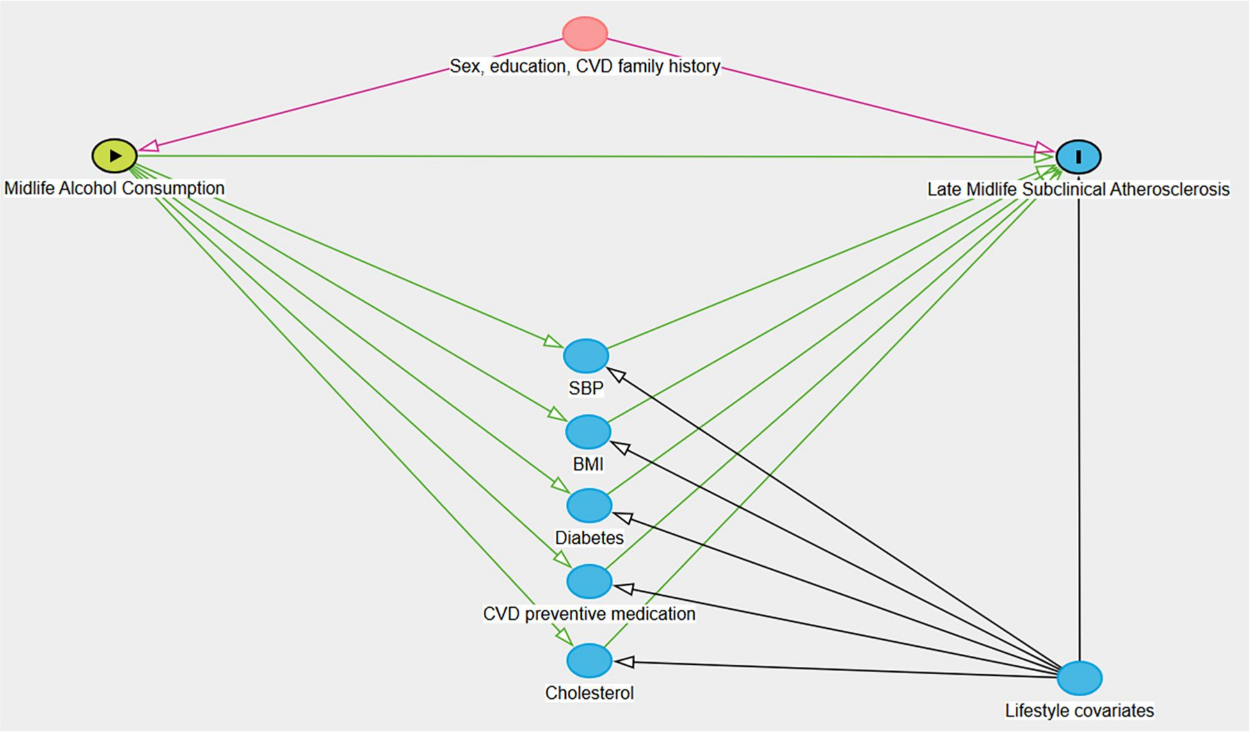

FIGURE S1
DAG illustrating potential causal relationship and outlining possible confounders in the association between midlife alcohol consumption and subclinical atherosclerosis. The DAG includes key cardiovascular lifestyle risk factor covariates (smoking, snus use, dietary pattern, and physical activity), mediators [BMI, systolic blood pressure (SBP), cholesterol, diabetes, and use of blood pressure- or lipid-lowering medication], and primary factors affecting both exposure and outcome (sex, educational level, and CVD family history).

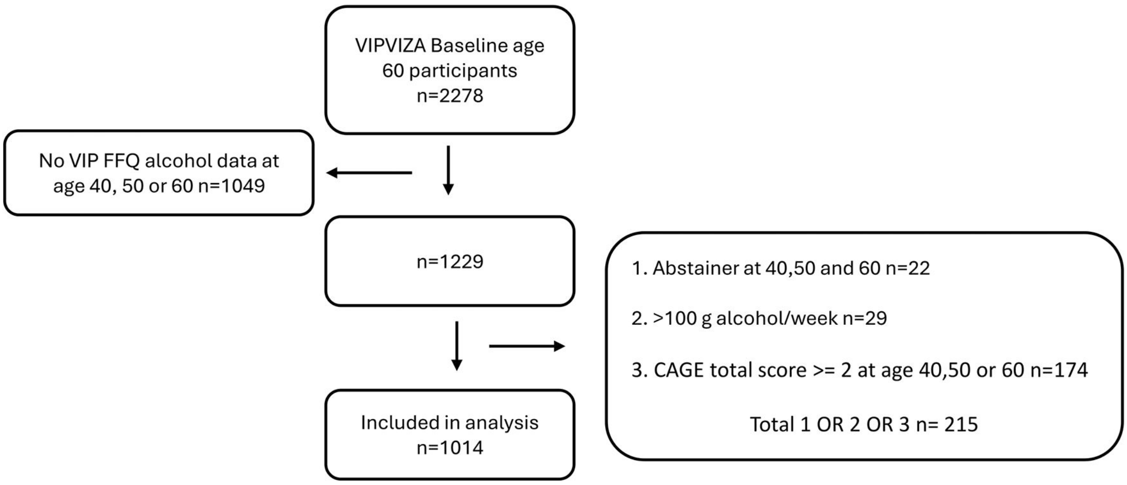

FIGURE S2
Exclusion procedure. VIP, Västerbotten intervention program m e;VIPVIZA, Visualisation of asymptomatic atherosclerotic disease for optimum cardiovascular prevention; FFQ, food frequency questionnaire; CAGE, cut down, annoyed, guilty, eye-opener.

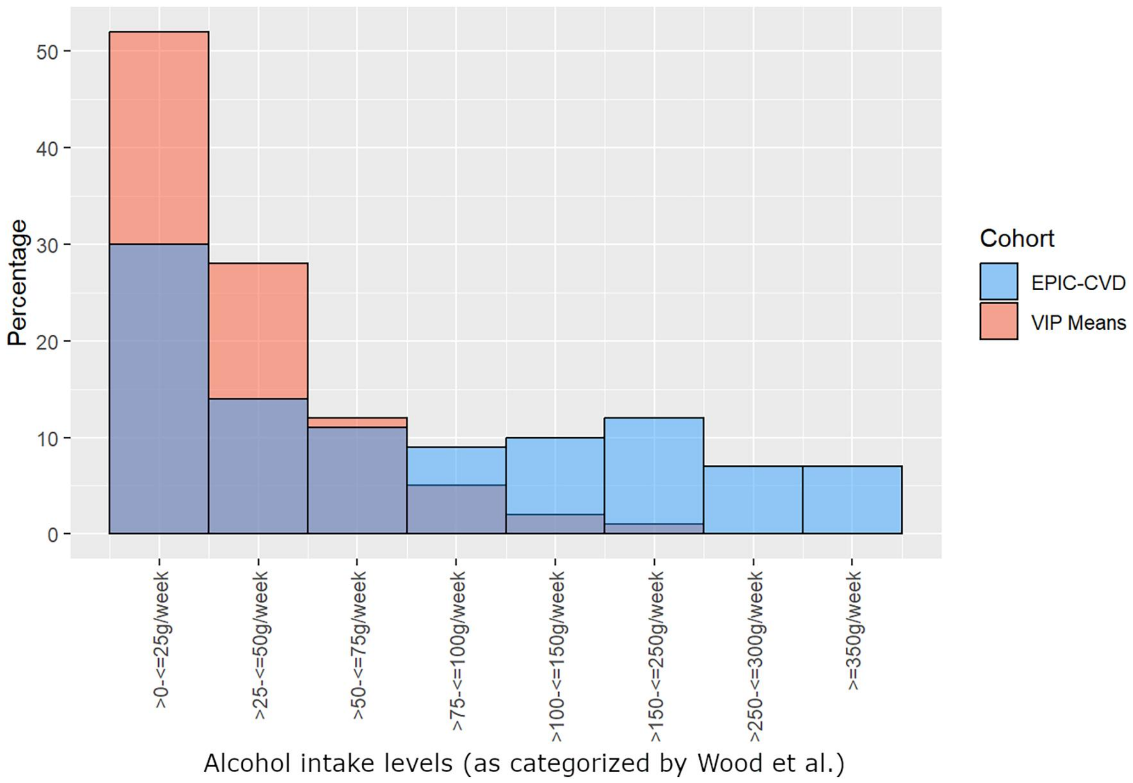

FIGURE S3
EPIC-CVD (n = 26 036) known current drinkers at baseline, alcohol consumption as reported by Wood et al. (18), histogram with staples representing percentage of cohort by grams of alcohol/week, with the VIP/VIPVIZA mean self-reported alcohol consumption/week for ages 40, 50, and 60 longitudinal cohort (n = 1207) overlapping for comparison.

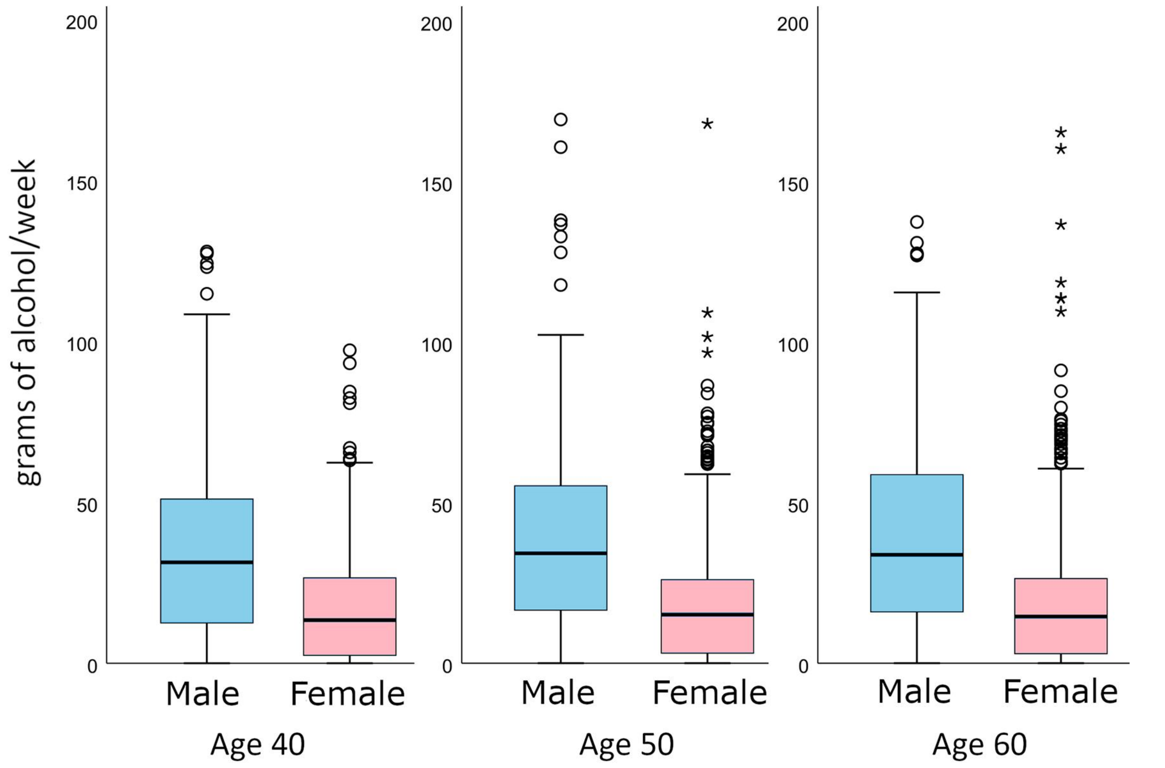

FIGURE S4
Boxplots displaying weekly alcohol consumption in grams, as reported by men and women at their VIP visit at ages 40, 50, and 60, only cases included in the analysis (n = 1,014) are shown.

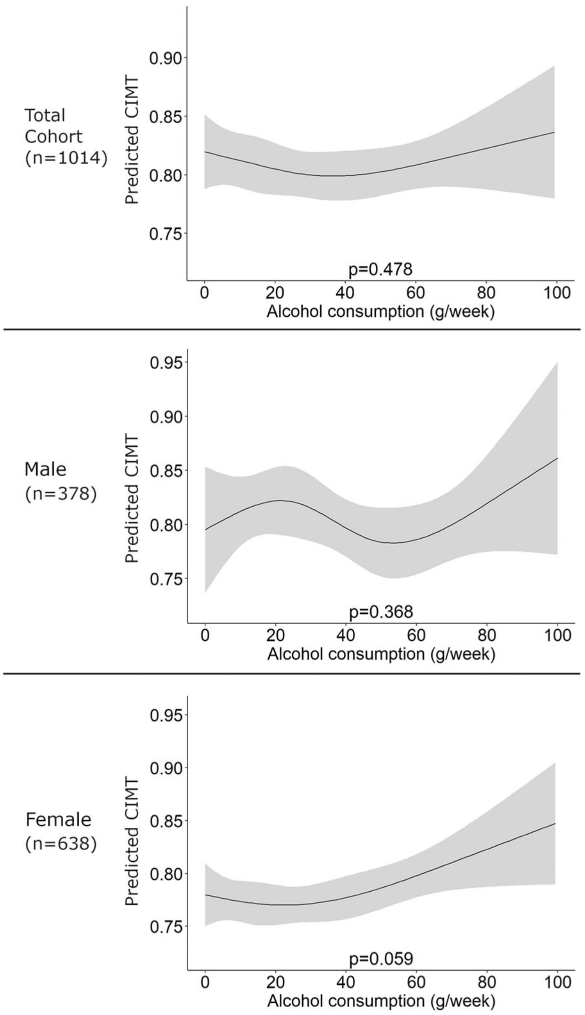

FIGURE S5

Association between mean weekly alcohol consumption (g/week) at ages 40, 50, and 60 and CIMT, modelled using restricted cubic splines with four knots. The upper panel shows the association for the total sample, while the middle and lower panels display results stratified by sex. The solid line represents the estimated association, and the shaded area depicts the 95% CI and *p* values, adjusted for model 2 covariates.
